# Cannabinoid receptor 2 facilitates the Schwann cells‐dependent peripheral nerve regeneration

**DOI:** 10.1002/ctm2.70184

**Published:** 2025-01-08

**Authors:** Heng Xu, Lu Wen, Yi Luo, Jiaying Zhou, Sheng Yao, Wei Ding, Jing Feng

**Affiliations:** ^1^ Department of Plastic and Reconstructive Surgery, Shanghai Ninth People's Hospital Shanghai Jiao Tong University School of Medicine Shanghai China; ^2^ Shanghai Key Laboratory of Tissue Engineering, Shanghai Ninth People's Hospital Shanghai Jiao Tong University School of Medicine Shanghai China; ^3^ Department of Burn and Plastic Surgery, Ruijin Hospital Shanghai Jiao Tong University School of Medicine Shanghai China; ^4^ State Key Laboratory of Chemical Biology, Shanghai Institute of Materia Medica Chinese Academy of Science Shanghai China; ^5^ School of Chinese Materia Medica Nanjing University of Chinese Medicine Nanjing China; ^6^ Zhongshan Institute for Drug Discovery, Shanghai Institute of Materia Medica Chinese Academy of Sciences Zhongshan China; ^7^ University of Chinese Academy of Sciences Beijing China

1

Dear Editor:

Here, we demonstrated that cannabinoid receptor 2 (CB2) plays a pivotal role in Schwann cells (SCs) by promoting the remyelination process following peripheral nerve injury (PNI). Selective activation of CB2 shows potential as a therapeutic approach to enhance nerve repair in these injuries.

Schwann cells form Büngner bands to guide axonal regeneration during PNI. However, the slow pace of axonal growth (∼1–3 mm/day) often leads to the degradation of these structures within 8 weeks, thereby disrupting the regenerative process.^1^, ^2^ CB2 activation has been shown to promote remyelination and enhance nerve regeneration by modulating inflammatory responses and supporting cellular processes essential for nerve repair.^3^, ^4^ However, the cellular mechanisms of CB2 function in remyelination remain unclear. This prompted the hypothesis that selective activation of CB2 could maintain Schwann cell function and create a more conducive environment for axonal regeneration.

To investigate this, we employed a mouse model of crush injury and treated the mice with delta‐9‐tetrahydrocannabinol (Δ^9^‐THC, a partial agonist of both CB1 and CB2 receptors, for 16 consecutive days; Figure S1A). Interestingly, Δ^9^‐THC treatment resulted in significantly increased growth rate of injured axons and improved behaviour in mice, including reflexive mechanical allodynia and walking parameters (Figure S1B–K). To exclude the possibility that Δ^9^‐THC exerts its effect via CB1 activation, we used CB2‐specific agonists. Although the agonist (GW842166X, EC50: ∼63 nM) did not improve mechanical pain (Figure S2A), the more efficient agonist, AM1241 (EC50: ∼3.4 nM), showed therapeutic efficacy. As expected, compared with vehicle treatment, daily administration of AM1241 significantly improved mechanical allodynia, gait abnormalities and motor function in mice. These improvements were further abolished when AM1241 was co‐administered with the CB2 antagonist AM630 (Figure 1 and Figure S2B). Consistent with these behavioural data, immunofluorescence staining showed that while nerves in the control group began to extend along their original growth direction, this change appeared to be more pronounced after AM1241 treatment (Figure S3A–C). We also examined the mRNA expression of genes related to myelination in the sciatic nerve tissues using real‐time quantitative PCR (RT‐qPCR). In the presence of AM1241, pro‐myelination‐related genes including Sox10, Egr2 and Tprv4 were significantly upregulated, whereas AM630 blocked CB2‐dependent gene upregulation. Trpv4 has been proved to delay the re‐myelination after nerve injury.^5^, ^6^ Interestingly, the expression of the SCs dedifferentiation‐related genes Sox2 and c‐Jun was not affected (Figure S3D). Moreover, Aniline Blue staining and electron microscopy demonstrated that CB2 stimulation significantly increased myelin thickness, an effect abolished by the CB2 antagonist AM630 (Figure S4). Supporting this, immunofluorescence staining and RT‐qPCR analyses of Mpz and Mbp showed consistent changes (Figure S5). Collectively, these findings suggest that pharmacological activation of CB2 enhances the promyelination process in Schwann cells, actively promoting nerve regeneration.

**FIGURE 1:**
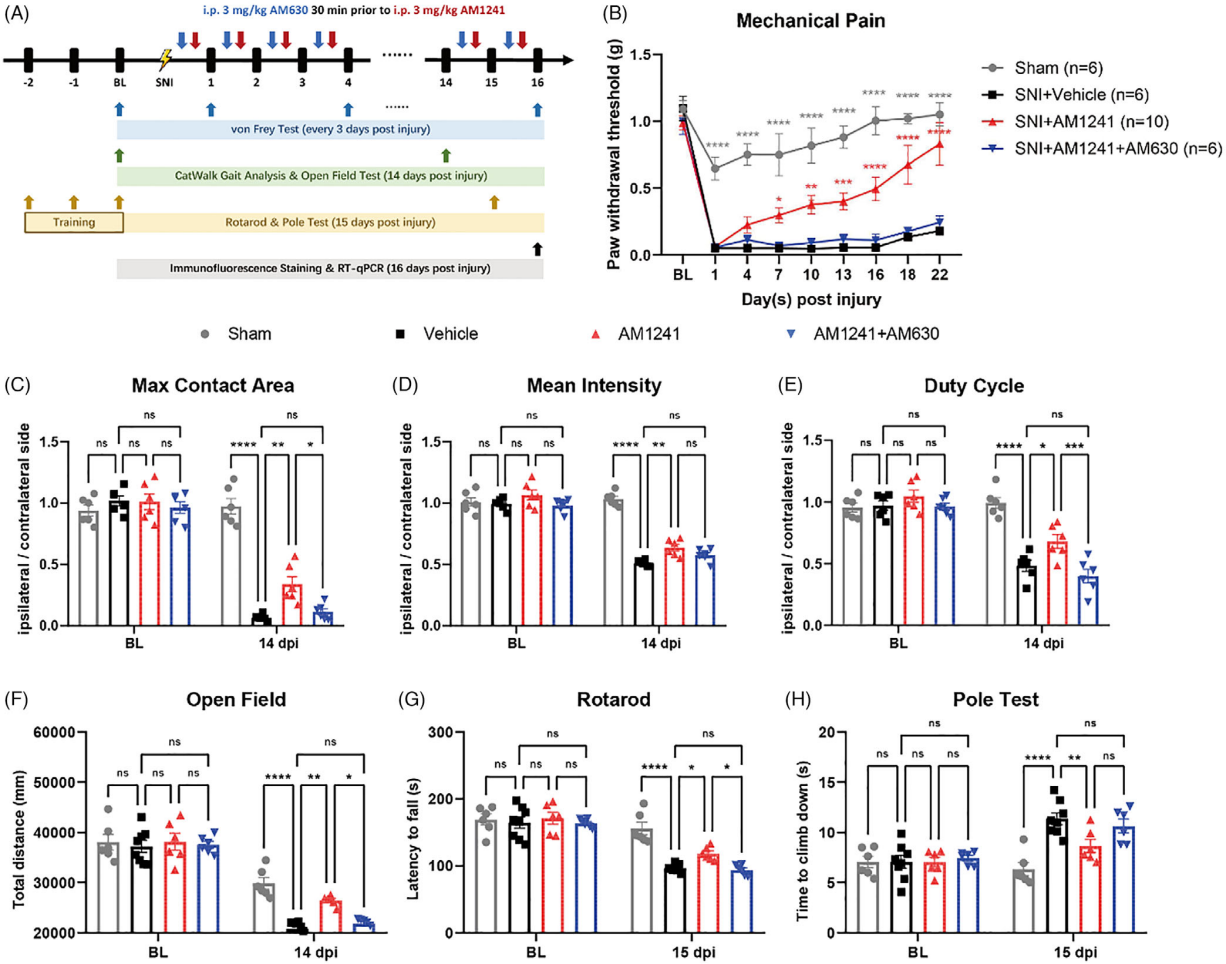
CB2 effects on function recovery in mice following SNI. (A) Schematic representation mice model setting up, including SNI surgery, drug treatment and behaviour assessment. (B) Time course of paw withdrawal threshold following sham or SNI surgery in mice with 16 days continuous treatment of AM1241 or AM630. *n* = 6–10 mice per group. Statistics were determined by two‐way analysis of variance (ANOVA). (C–E) CatWalk gait analysis on day 14 after surgery, including (C) max contact area, (D) mean intensity and (E) duty cycle. *n* = 6 mice per group. Statistics were determined by one‐way ANOVA. (F–H) Motor function analysis on day 14 and 15 after surgery, including (F) open field, (G) rotarod and (H) pole test. *n* = 6–8 mice per group. Statistics were determined by one‐way ANOVA. ns: no significance, *p < .05, **p < .001, ***p < .005, ****p < .0001. SNI, sciatic nerve injury.

As CB2 is widely distributed in the periphery, we investigated the specific role of SC‐expressing CB2 in nerve repair. To test this hypothesis, we crossed Cnr2 (the gene encoding CB2) floxed mice with mice expressing Cre recombinase under the control of the Plp1 promoter to generate mice lacking Cnr2 in SCs. After tamoxifen treatment, an sciatic nerve injury (SNI) model was established in *Cnr2^F/F^::Plp1^creERT^* mice and their littermates (Figure 2A). Interestingly, behavioural tests showed that the paw withdrawal threshold of *Cnr2^F/F^* mice returned to normal levels approximately 10 weeks after surgery, whereas *Cnr2^F/F^::Plp1^creERT^* mice were still experiencing SNI‐induced pain (Figure 2B). Gait analysis (Figure 2C–F) and motor function tests (Figure 2G–I) also confirmed the delayed functional recovery after genetic ablation of CB2 in SCs. Moreover, *Cnr2^F/F^::Plp1^creERT^* mice showed lower fluorescence intensity of AQP1, MPZ, MBP and sporadically distributed TUJ1 signals when compared with that in the *Cnr2^F/F^* mice (Figures S6 and S7). These data suggest that SC‐expressed CB2 is essential for peripheral nerve regeneration.

**FIGURE 2:**
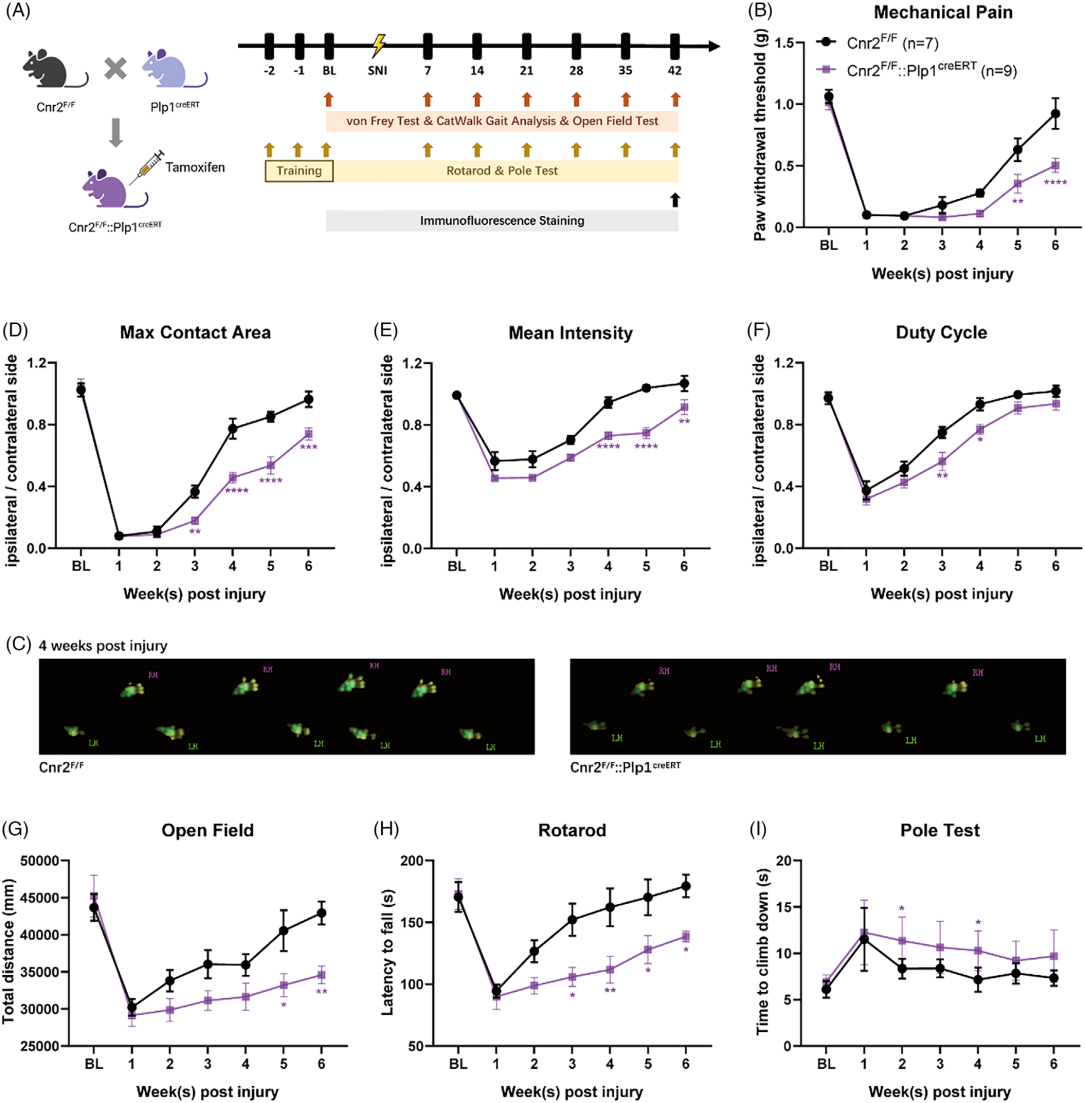
CB2 in Schwann cells (SCs) contributes to function recovery in mice. (A) Schematic diagram of breeding *Cnr2^F/F^::Plp1^creERT^* mice, and setting up model, including SNI surgery, drug treatment and behaviour assessment. (B) Time course of paw withdrawal threshold following SNI surgery in *Cnr2^F/F^::Plp1^creERT^* and *Cnr2^F/F^* mice during 6 weeks. *n* = 7–9 mice per group. Statistics were determined by two‐way analysis of variance (ANOVA). (C–E) Time course of CatWalk gait analysis in 6 weeks, including (C) max contact area, (D) mean intensity and (E) duty cycle. (F) Representative timing view of fluorescence visible footprint in *Cnr2^F/F^::Plp1^creERT^* and *Cnr2^F/F^* mice on the fourth week after surgery. (G–I) Time course of motor function analysis in 6 weeks, including (G) open field, (H) rotarod and (I) pole test. *p < .05, **p < .001, ***p < .005, ****p < .0001.

To clarify the role of SC‐expressing CB2, we first confirmed that Cnr2 is substantially expressed in SCs by RT‐qPCR, whereas Cnr1 showed much lower expression (Figure 3A). As a result, pharmacological activation of CB2 with AM1241 upregulated the expression of myelination‐related factors, which was reversed by the co‐application of AM630 (Figure 3B). We then determined the CB2 function using in vitro calcium imaging on cultured primary SCs isolated from newborn mice. Interestingly, perfusion with AM1241 elicited robust calcium influx in SCs, which were nearly abolished by the co‐application of the CB2 antagonist AM630 (Figure 3C–E). Moreover, the Ki67 positive signal also significantly increased upon AM1241 stimulation, and the proportion of Ki67^+^/SOX10^+^ cells was reduced by pre‐incubation with AM630 (Figure 3F,G). Consistently, the number of viable cells determined using the cell counting kit‐8 showed that the activation of CB2 promoted cell proliferation (Figure 3H). Taken together, these results suggest that CB2 functionally regulates SCs proliferation, and the activation of SCs‐expressing CB2 may accelerate injured peripheral nerve re‐myelination.

**FIGURE 3:**
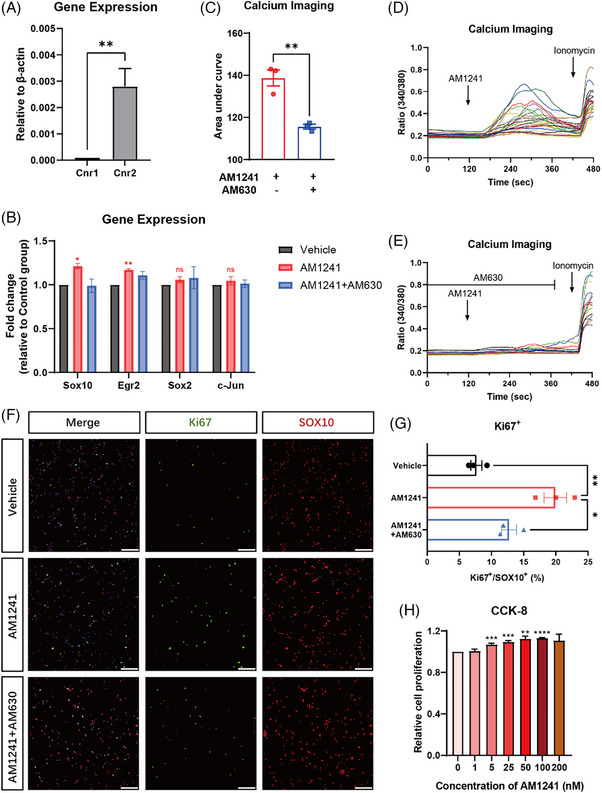
Functional expression of CB2 in Schwann cells (SCs). (A) Relative expression levels of Cnr1 and Cnr2 mRNA in mouse SCs. *n* = 4 samples from eight mice per group. Statistics were determined by unpaired two‐tailed *t*‐test. (B) Relative expression levels of myelination‐related proteins (Sox10, Egr2, Sox2, and c‐Jun) in mouse SCs with incubation of AM1241 and AM1241+AM630. *n* = 3 samples from six mice per group. Statistics were determined by one‐way analysis of variance (ANOVA). (C–E) Representative time‐lapse traces of average [Ca^2+^]_i_ responses to AM1241 (100 nM) and co‐application with AM630 (100 nM) in mouse SCs, and quantification of the area under curve. *n* = 3 slices from six mice per group. Statistics were determined by unpaired two‐tailed *t*‐test. Representative image of (F) with proliferation marker Ki67 and SOX10 antibody in primary mouse SCs with incubation of AM1241 and AM1241+AM630, and (G) quantification of the proportion of Ki67^+^/SOX10^+^ SCs. Scale bars: 100 µm. *n* = 3 slices from six mice per group. Statistics were determined by one‐way ANOVA. (H) Relative cell proliferation of primary mouse SCs with AM1241 treatment measured by cell counting kit‐8 (CCK‐8) assay. Statistics were determined by unpaired two‐tailed *t*‐test. ns: no significance, *p < .05, **p < .001, ***p < .005, ****p < .0001.

Resident macrophages facilitate axonal sprouting into the distal stump by clearing debris,^7^ and macrophage‐expressed CB2 may play a potential role in this process and promote peripheral nerve repair.^8^ To investigate whether CB2 expressed in macrophages contributes to this process, we genetically ablated CB2 from SCs by crossing the *Cnr2^F/F^* with *CX3CR1^creERT^* mice (Figure S8A). After the induction of SNI model, behavioural tests revealed no significant differences between the *Cnr2^F/F^:: CX3CR1^creERT^* mice and control littermates (Figure S8B–I), suggesting that resident macrophage‐expressed CB2 may be not involved in peripheral nerve repair.

SCs are critical for forming bands of Büngner, which serve as essential structural and molecular guides for axonal regeneration following PNI.^9^, ^10^ Enhancing the activity of SCs could substantially improve the efficiency of nerve regeneration. In this study, we demonstrated that pharmacological activation of CB2 markedly enhanced SC proliferation and promyelination, while genetic ablation of SC‐specific CB2 aggravated pain and motor dysfunction in the SNI model (Figure S9). These findings highlight the therapeutic potential of targeting SC‐expressed CB2 to promote remyelination and improve recovery outcomes after nerve injury.

## AUTHOR CONTRIBUTIONS

Heng Xu designed research, performed research and wrote the paper. Lu Wen performed research, analyzed data and wrote the paper. Yi Luo performed research and analyzed data. Jiaying Zhou performed research. Sheng Yao performed research and contributed new reagents. Wei Ding contributed new reagents or analytic tools. Jing Feng designed research, contributed new reagents or analytic tools and wrote the paper.

## CONFLICT OF INTEREST STATEMENT

The authors declare no conflicts of interest.

2

## Supporting information



Supporting information

## Data Availability

The data that support the findings of this study are available from the corresponding author upon reasonable request. Some data may not be made available because of privacy or ethical restrictions.
