# Supplementary material for: Cannabinoid receptor 2 facilitates the Schwann cells‐dependent peripheral nerve regeneration
Source: Clin Transl Med. 2025 Jan 8;15(1):e70184. doi: 10.1002/ctm2.70184 (PMC11707426; doi:10.1002/ctm2.70184)
Supplement: Supplementary file 1 — Supporting information [file CTM2-15-e70184-s001.docx]

**Supplementary**

**Methods and Materials**

**Animals** Adult male and female mice of 8-12 weeks weighting 20-25 g were used in behavior test. C57BL/6JGpt mice were purchased from GemPharmatech Co., Ltd (Nanjing, China). Cnr2-flox mice (Stock No: S-CKO-01791) were purchased from Cyagen Biosciences Co., Ltd (Suzhou, China). Plp1-creERT (Stock No: 005975) and Cx3cr1-creERT mice (Stock No: 021160) were purchased from The Jackson Laboratory. Mice were housed in specific pathogen-free barrier facilities (temperature: 24±2℃, humidity: 50±10%) under 12/12-h light/dark cycles with free access to food and water. All animal experiments were approved by Institutional Animal Care and Use Committees (IACUC), Shanghai Institute of Materia Medica, Chinese Academy of Sciences.

**Drug administration** The following drugs with corresponding doses were used for mice: AM1241 (TargetMol #T6755, 3 mg/kg in 0.8% DMSO and 1% Tween 80) and AM630 (Macklin #A864284, 3 mg/kg in 0.8% DMSO and 1% Tween 80). Δ^9^-THC (10 mg/kg in 0.2% DMSO and 1% Tween 80) was provided by State Key Laboratory of Drug Research and Natural Products Chemistry Department, Shanghai Institute of Materia Medica. Intraperitoneal administration of tamoxifen for 5 consecutive days at 100 mg/kg in corn oil was used to for the induction of cre activity in inducible cre-driver lines. As a supplement, tamoxifen was administrated at the same dose every three days from the modeling day until the ending point.

**Sciatic nerve injury** Mice was anesthetized with isoflurane and the skin and muscle of the left thigh were incised to explore sciatic nerve. After exploration, the never was crushed for 15 sec by hemostatic forceps, locking the ratchet at the first position. The skin was stitched and disinfected with betadine. The sham surgery was the same as above procedure, but the nerve was not crushed.

**Behavior test** The von Frey test was conducted by exposing the plantar surface of the hind-paw to a series of 10 von Frey filaments. Starting with the 0.4 g filament, von Frey filaments ranging from 0.04 to 2 g bending force were applied, using the up-down method to determine threshold sensitivity. CatWalk gait analysis was evaluated by CatWalk™ XT Version 10.5 (Noldus). Data was represented as ipsi (ipsilateral, left-hind paw) / contra (contralateral, right-hind paw). Animal behavior analysis system developed by Xinruan Information Technology Co., Ltd (Shanghai, China) was used to record the performance in open field. The accelerating rotarod (IITC) was setting at 5–40 rpm with a cut-off time of 300 sec. The pole was with a height of 55 cm and a top diameter of 8 mm. The cut-off time of pole test was 20 sec in both T-turn and T-down.

**Immunofluorescence staining** Tissues and cells were dissected and post-fixed with 4% (wt/vol) paraformaldehyde overnight (for tissue) or 15 min (for cell) at 4℃. Following, tissues were dehydrated in 30% (wt/vol) sucrose for 1 day at 4℃ and embedded in Tissue-Tek® O.C.T. Compound (Sakura). Cryosections were cut at 12 µm. Tissue sections and cells were permeabilized and blocked in 0.3% Triton X-100 (J&K) and 10% (vol/vol) normal goat serum in PBS for 2 h, then incubated overnight at 4℃ with the following primary antibodies: AQP1 (Santa Cruz #sc-25287, 1:400), TUJ1 (BioLegend #802001, 1:400), Ki67 (abcam #ab15580, 1:500), SOX10 (Santa Cruz #sc-365692, 1:200), Myelin Basic Protein (MBP) (abcam #ab40390, 1:500) and Myelin Protein Zero (MPZ) (Aves Labs Cat# PZ0-0020,1:300). After washing 3 times with PBS, sections and cells were stained with the corresponding secondary antibodies for 2 h at RT. Secondary antibodies used are: Alexa Fluor® 488 AffiniPure™ Donkey Anti-Mouse IgG (H+L) (Jackson #715-545-151, 1:300), Cy™3 AffiniPure Donkey Anti-Rabbit IgG (H+L) (Jackson #711-165-152, 1:300), Alexa Fluor® 488 AffiniPure™ Donkey Anti-Rabbit IgG (H+L) (Jackson #711-545-152, 1:300), Cy™3 AffiniPure Donkey Anti-Mouse IgG (H+L) (Jackson #715-165-151, 1:300) and Alexa Fluor® 488 AffiniPure™ Goat Anti-Chicken IgY (H+L) (abcam #ab150169, 1:300). All sections and cells were imaged using a Leica inverted confocal microscope (SP5 model, Wetzlar, Germany). Integrated density and fluorescent area occupied by SCs and axon within the nerve bridge (region between two stumps) was quantified using ImageJ. Cell count was performed with ImageJ.

**Morphological analyses** Sciatic nerves distal to the injury area were rapidly removed, cut in small segments (3 mm length), and fixed by immersion in 4% formaldehyde, 2.5% glutaraldehyde for 12h at 4 °C. Tissue samples were washed in phosphate buffer and postfixed for 90 min at room temperature in 1% OsO4 and then dehydrated starting in an ethanol series and ending with propylene. Tissue samples were then embedded in a mixture of resins. The samples were oriented longitudinally and embedded in Epon. Nerves were sectioned transversely using an ultramicrotome. Semi-thin sections (1 mm) stained with toluidine blue were prepared for evaluation under a light microscope (Primo Star, Carl Zeiss AG). Ultrathin (90 nm) sections were stained with 3% uranyl acetate-lead citrate and then observed under transmission electron microscopy (HT7800, Hitachi High-Technologies) and photographed.

**Real-time quantitative PCR** RNA was extracted using TRIzol™ reagent (Invitrogen) and reversetranscribed into cDNA using HyperScriptⅢ RT SuperMix for qPCR with gDNA Remover kit (EnzyArtisan) according to the manufacturer’s instructions. The expression of mRNAs was determined using ChamQ Universal SYBR qPCR Master Mix (Vazyme). The relative gene expression was calculated using the comparative CT method. β-actin was used as an internal control to normalize gene expression. The sequences of the primers used are as follows (5’→3’):

β-actin forward GTGACGTTGACATCCGTAAAGA, reverse GCCGGACTCATCGTACTCC;

Cnr1 forward AAGTCGATCTTAGACGGCCTT, reverse TCCTAATTTGGATGCCATGTCTC;

Cnr2 forward ATGGCCGTGCTCTATATTATCCT, reverse ATGGTCACACTGCCGATCTTC;

Sox10 forward CGGACGATGACAAGTTCCCC, reverse GTGAGGGTACTGGTCGGCT;

Egr2 forward GCCAAGGCCGTAGACAAAATC, reverse CCACTCCGTTCATCTGGTCA;

Sox2 forward GCGGAGTGGAAACTTTTGTCC, reverse GGGAAGCGTGTACTTATCCTTCT;

c-Jun forward TTCCTCCAGTCCGAGAGCG, reverse TGAGAAGGTCCGAGTTCTTGG.

MBP forward ATTCACCGAGGAGAGGCTGGAA, reverse TGTGTGCTTGGAGTCTGTCACC;

MPZ forward CTGCTCCTTCTGGTCCAGTGAA, reverse AGGTTGTCCCTTGGCATAGTGG;

Trpv4 forward TCACCGCCTACTATCAGCCACT, reverse GAACAGGACTCCTGTGAAGAGC.

**Primary mouse Schwann cell** Primary mouse SCs from sciatic nerves of newborn mice (0-3 d old) were isolated and cultured as described previously[11].

**Calcium imaging** Live cell calcium (Ca^2+^) imaging was performed on cultured primary mouse Schwann cell loaded with calcium indicator Fura-2-acetoxymethyl ester (Fura-2 AM, 4 μM) for 60 min at 37°C. Before experiment, Fura-2-loaded cells were washed with HBSS for 2 times. The bath solution contained (in mM): 140 NaCl, 5 KCl, 2 CaCl_2_, 2 MgCl_2_, 10 HEPES, 30 sucrose and 10 glucose, adjusted to pH 7.3 with NaOH. Radiometric calcium imaging was performed using a Leica inverted confocal microscope (SP5 model, Wetzlar, Germany) at room temperature. Fura-2 ratios (F_340_/F_380_) reflecting changes in the intracellular concentrations of calcium [Ca^2+^]_i_ upon AM1241 (100 nM) or AM630 (100 nM) were monitored and recorded. The viability of cell was confirmed by an increase in [Ca^2+^]_i_ elicited by ionomycin (1 μM) perfusion at the end of the experiment. Cells were considered responsive if an increase in fluorescence ratio (F_340_/F_380_) was equal or greater than 20% above baseline.

**Statistical analysis** Data are represented as mean ± SEM. Statistical analysis was carried out using GraphPad Prism 9.0.0 software (GraphPad Software Ltd., San Diego, CA, USA). P < 0.05 was considered statistically significant, and indicated by asterisks as follows: * P < 0.05, ** P < 0.01, *** P < 0.001, **** P < 0.0001.

**Figure**

**
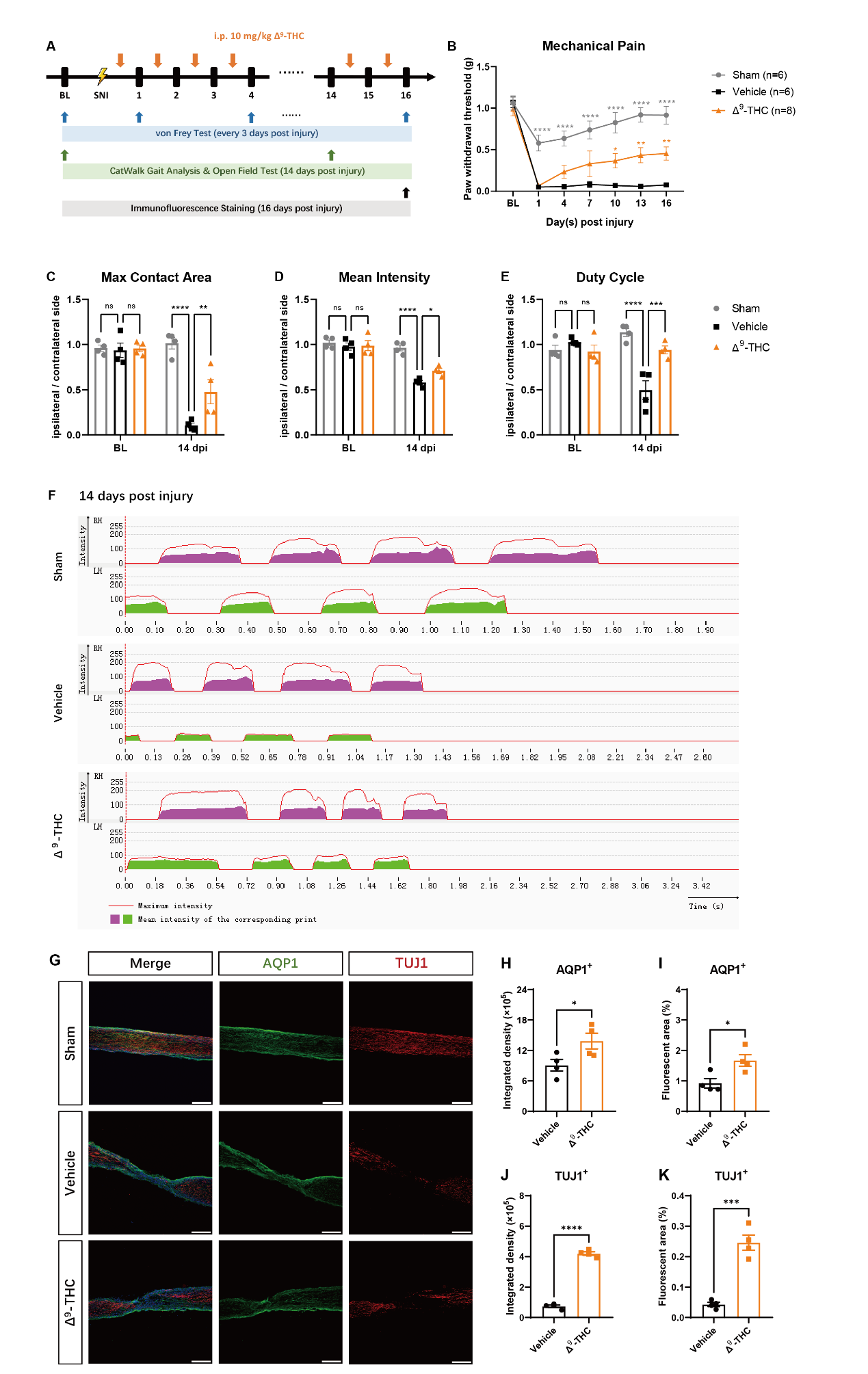
**

**FigS1. Δ^9^-THC promotes peripheral nerve injury repair**

**(A)** Schematic representation of mice model setting up, including SNI surgery, drug treatment and behavior assessments.

**(B)** Time course of paw withdrawal threshold following sham or SNI surgery in mice with 16 days continuous treatment of Δ^9^-THC. n = 6-8 mice per group. Statistics were determined by two-way ANOVA.

**(C-E)** CatWalk gait analysis on day 14 after surgery, including **(C)** max contact area, **(D)** mean intensity and **(E)** duty cycle. n = 4 mice per group. Statistics were determined by one-way ANOVA.

**(F)** Representative timing view of 2D footprint intensity on day 14 after surgery. ns: no significance.

**(G)** Representative IF staining by utilizing AQP1 and TUJ1 antibody in sciatic nerve section of sham, vehicle and Δ^9^-THC on the 6th week after SNI surgery. Scale bars: 100 μm.

**(H-I)** Quantified analysis of the **(H)** integrated density and **(I)** area of AQP1 at the injured area.

**(J-K)** Quantified analysis of the **(J)** integrated density and **(K)** area of TUJ1 at the injured area. n = 4 slices from 4 mice per group. Statistics were determined by unpaired two-tailed t test. *: p<0.05, **: p<0.001, ***: p<0.005, ****: p<0.0001.


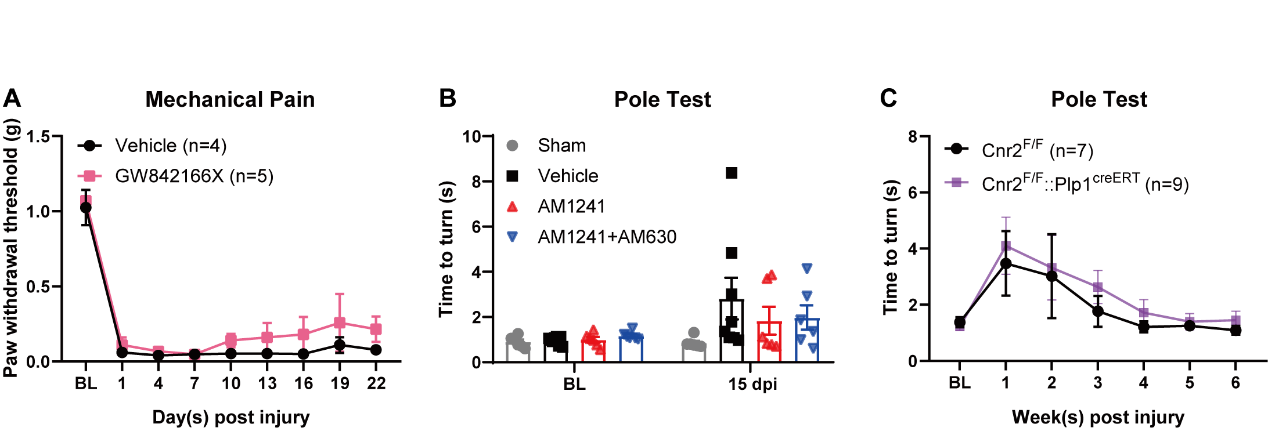


**FigS2. (A)** Using GW842166X in treating SNI models for 22 days and test the mechanical pain. n=4 mice in vehicle group and n=5 in GW842166X group. The two groups have no significant difference.

**(B)** Using AM1241 and AM630 in treating SNI models for 15 days to perform the pole test. n=4 mice per group. The four groups have no significant difference.


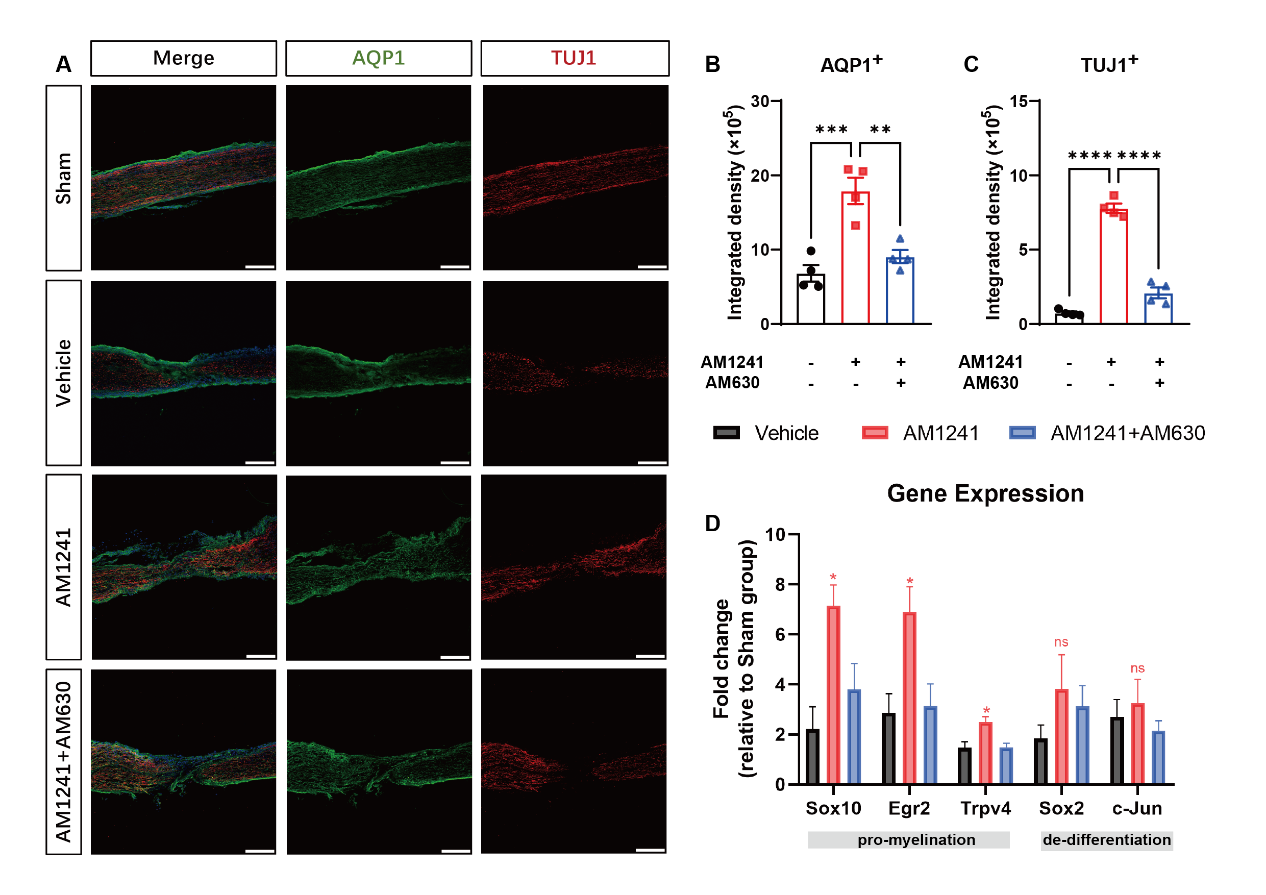


**FigS3. CB2 effects on nerve regeneration after injury**

**(A)** Representative IF staining by utilizing AQP1 (aquaporin 1) and TUJ1 (tubulin β-3) antibody in sciatic nerve section of mice with 16 days continuous treatment of AM1241 or AM630 after surgery. Scale bars: 100 μm.

**(B-C)** Quantified analysis of the integrated density of **(B)** AQP1^+^ ,**(C)** TUJ1^+^ in injured region. n = 4 slices from 4 mice per group. Statistics were determined by one-way ANOVA.

**(D)** Relative expression levels of myelination-related genes in injured sciatic nerve tissue. n = 3 samples from 3 mice per group. Statistics were determined by one-way ANOVA. ns: no significance, *: p<0.05, **: p<0.001, ***: p<0.005, ****: p<0.0001.


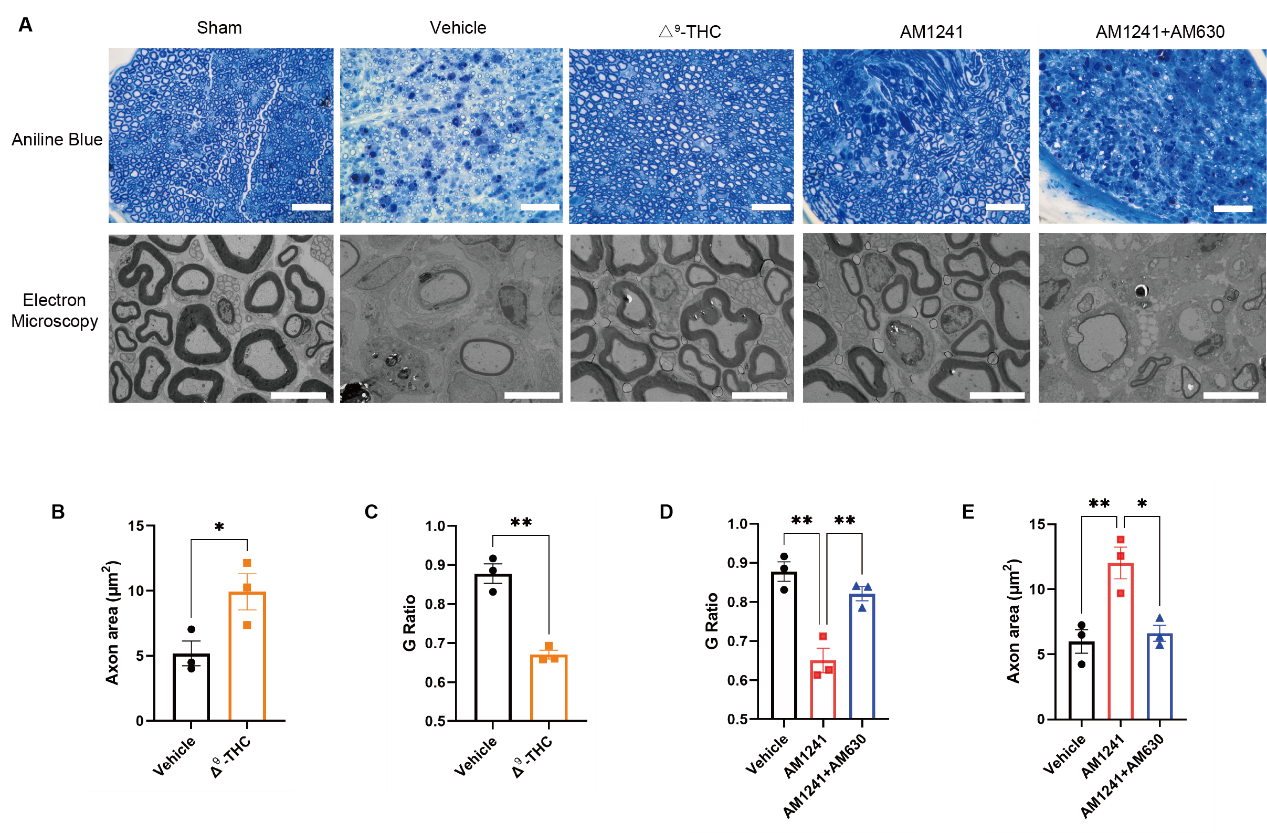


**FigS4. Pharmacological effects on myelination**

**(A)** Representative Aniline Blue staining and Electron Microscopy in sciatic nerve section of mice with 16 days continuous treatment of Δ^9^-THC, AM1241 or AM630 after surgery. Scale bars: 100 μm in Aniline Blue staining; 5 μm in Electron Microscopy.

**(B-E)** Quantified analysis of the Axon area and G Ratio of **(B-C)** Vehicle v.s. Δ^9^-THC, **(D-E)** comparing Δ^9^-THC, AM1241, AM1241+AM630. n = 4 slices from 4 mice per group. Statistics were determined by one-way ANOVA. *: p<0.05, **: p<0.001.


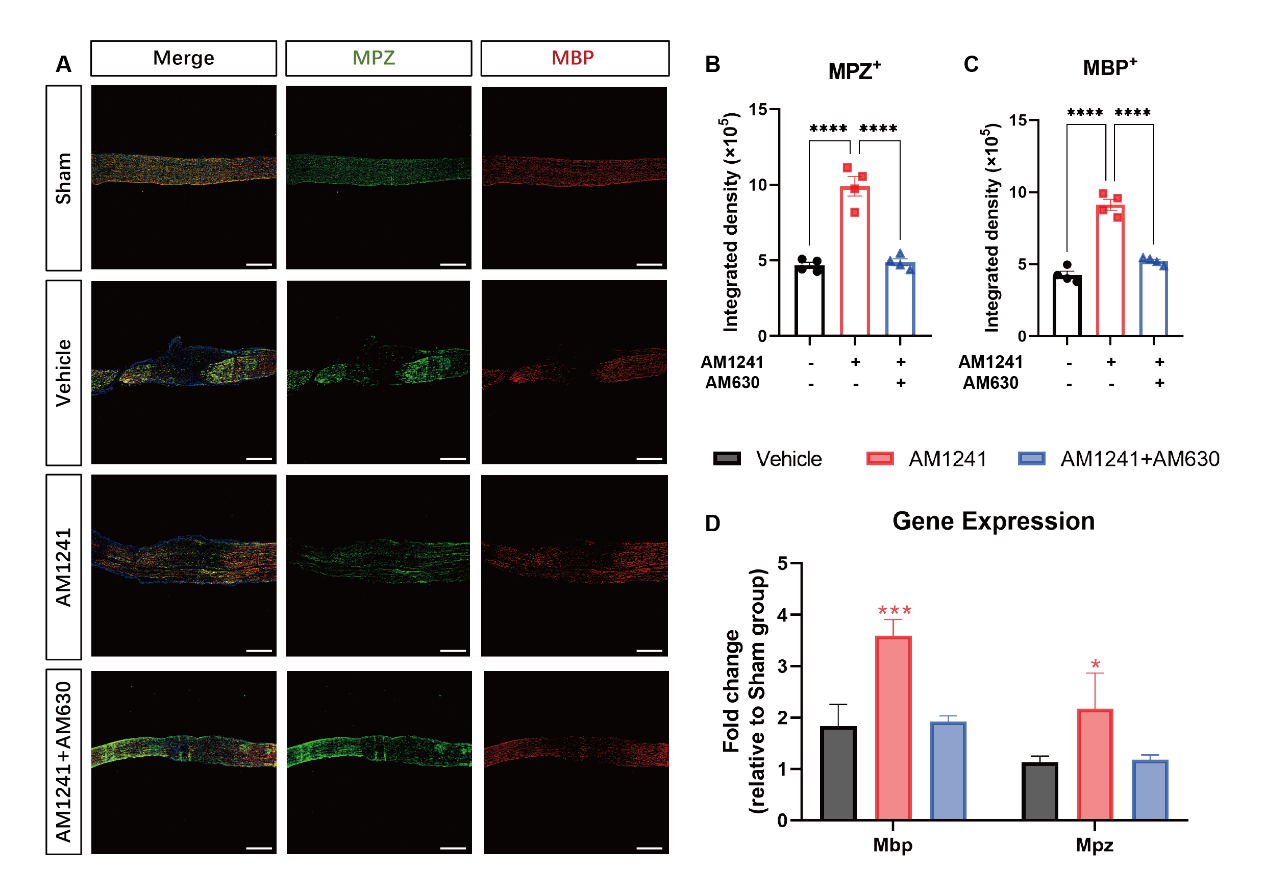


**FigS5. CB2 effects on myelination after injury**

**(A)** Representative IF staining by utilizing Mbp (myelin basic protein) and Mpz (myelin protein zero) antibody in sciatic nerve section of mice with 16 days continuous treatment of AM1241 or AM630 after surgery. Scale bars: 100 μm.

**(B-C)** Quantified analysis of the integrated density of (B) MBP+ ,(C) MPZ+ in injured region. n = 4 slices from 4 mice per group. Statistics were determined by one-way ANOVA.

**(D)** Relative expression levels of Mbp and Mpz genes in injured sciatic nerve tissue. n = 3 samples from 3 mice per group. Statistics were determined by one-way ANOVA.

*: p<0.05, ***: p<0.005, ****: p<0.0001.


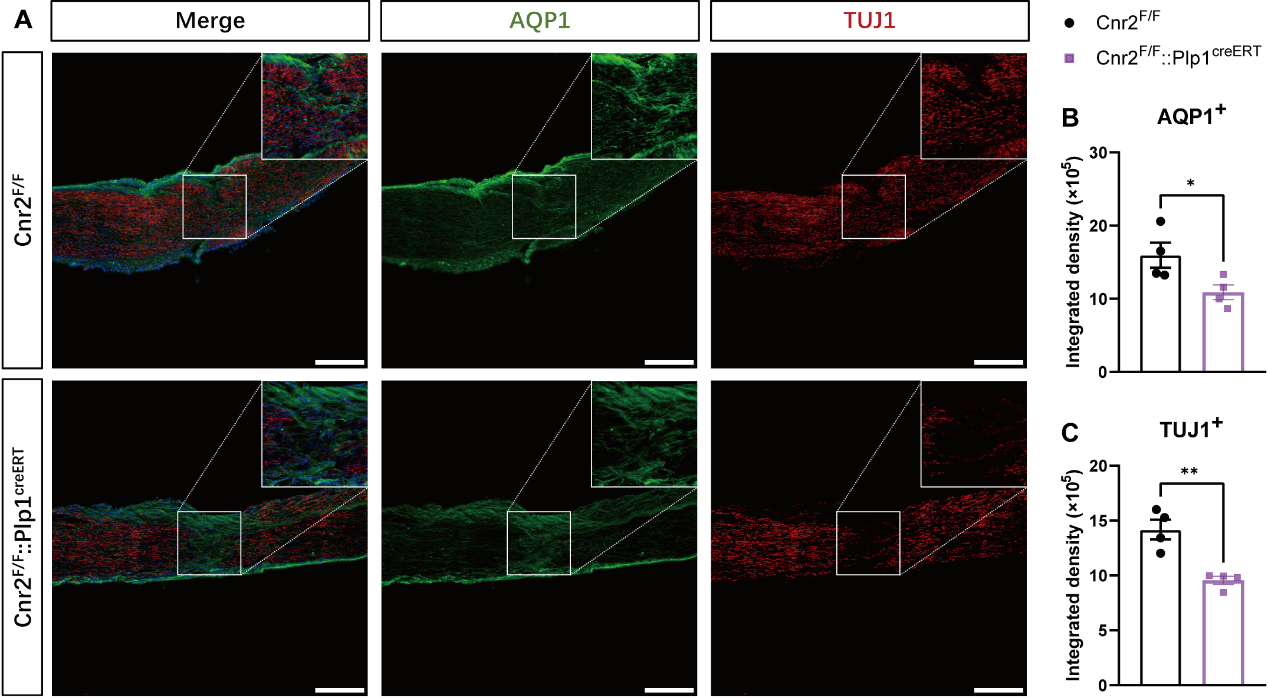


**FigS6. SCs-expressing CB2 is conducive to nerve regeneration**

**(A)** Representative IF staining by utilizing AQP1 and TUJ1 antibody in sciatic nerve section of Cnr2^F/F^::Plp1^creERT^ and Cnr2^F/F^ on the 6th week after SNI surgery. Scale bars: 100 μm.

**(B-C)** Quantified analysis of the integrated density and fluorescence area of **(B)** AQP1^+^ ,**(C)** TUJ1^+^ at the injured area. n = 4 slices from 4 mice per group. Statistics were determined by unpaired two-tailed t test. *: p<0.05, **: p<0.001.


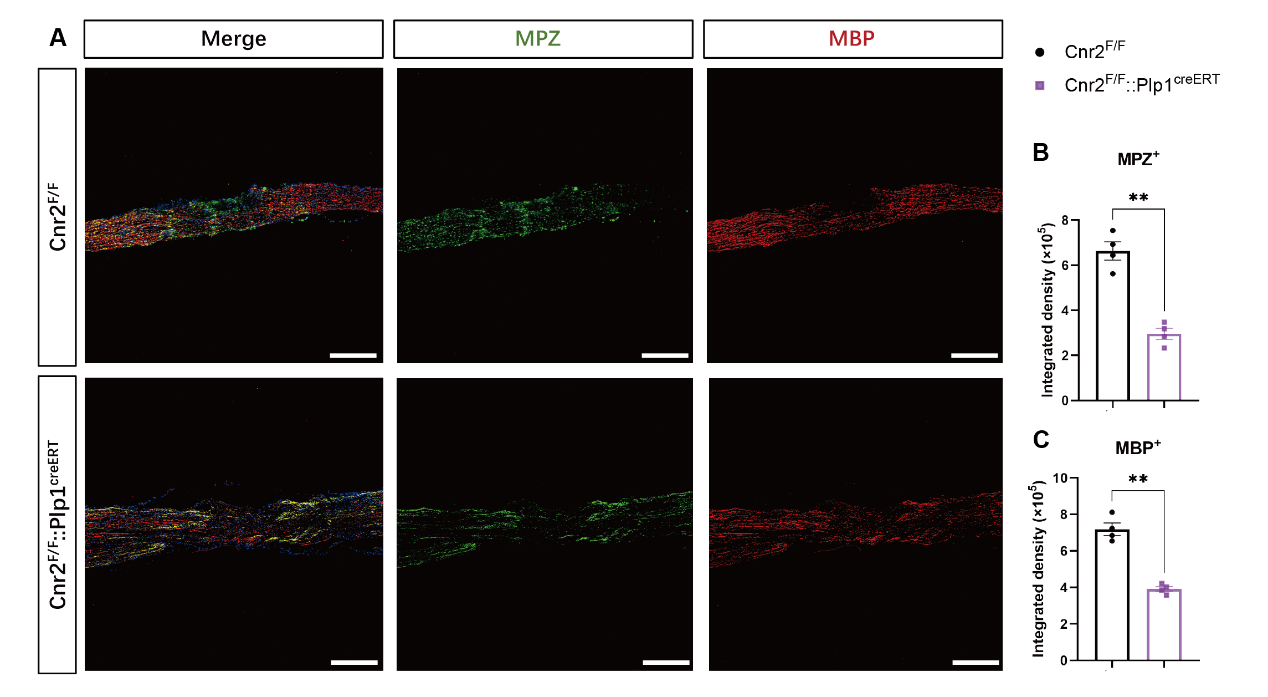


**FigS7. Myelin related markers in CB2 conditional deleted mice.**

**(A)** Representative IF staining by utilizing MPZ and MPB antibody in sciatic nerve section of Cnr2^F/F^::Plp1^creERT^ and Cnr2^F/F^ on the day 16 after SNI surgery. Scale bars: 100 μm.

**(B-C)** Quantified analysis of the integrated density of **(B)** MPZ^+^ ,**(C)** MBP^+^ at the injured area. n = 4 slices from 4 mice per group. Statistics were determined by unpaired two-tailed t test. *: p<0.05, **: p<0.001.


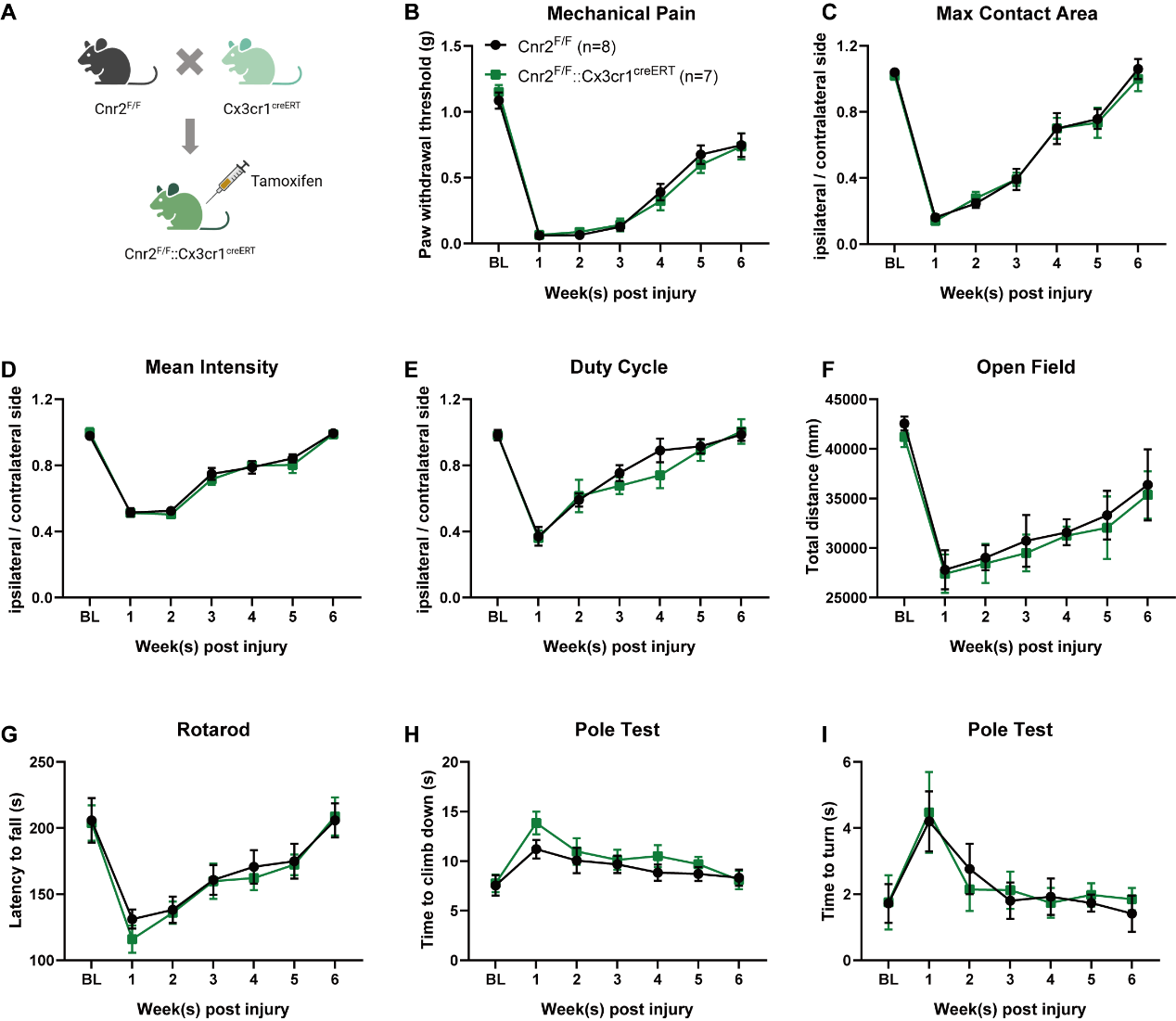


**FigS8. CB2 in CX3CR1+macrophage doesn’t effect the function recovery in mice**

**(A)** Schematic diagram of breeding Cnr2^F/F^::Cx3cr1^creERT^ mice, and setting up model, including SNI surgery, drug treatment and behavior assessment.

**(B)** Mechanical pain test following SNI surgery in Cnr2^F/F^::Cx3cr1^creERT^ and Cnr2^F/F^ mice during 6 weeks. n = 7-8 mice per group. Statistics were determined by two-way ANOVA.

**(C-E)** Time course of CatWalk gait analysis in 6 weeks, including **(C)** max contact area, **(D)** mean intensity and **(E)** duty cycle.

**(F-I)** Time course of motor function analysis in 6 weeks, including **(F)** open field, **(G)** rotarod and **(H-I)** pole test. All the results in this figure have no significant difference.


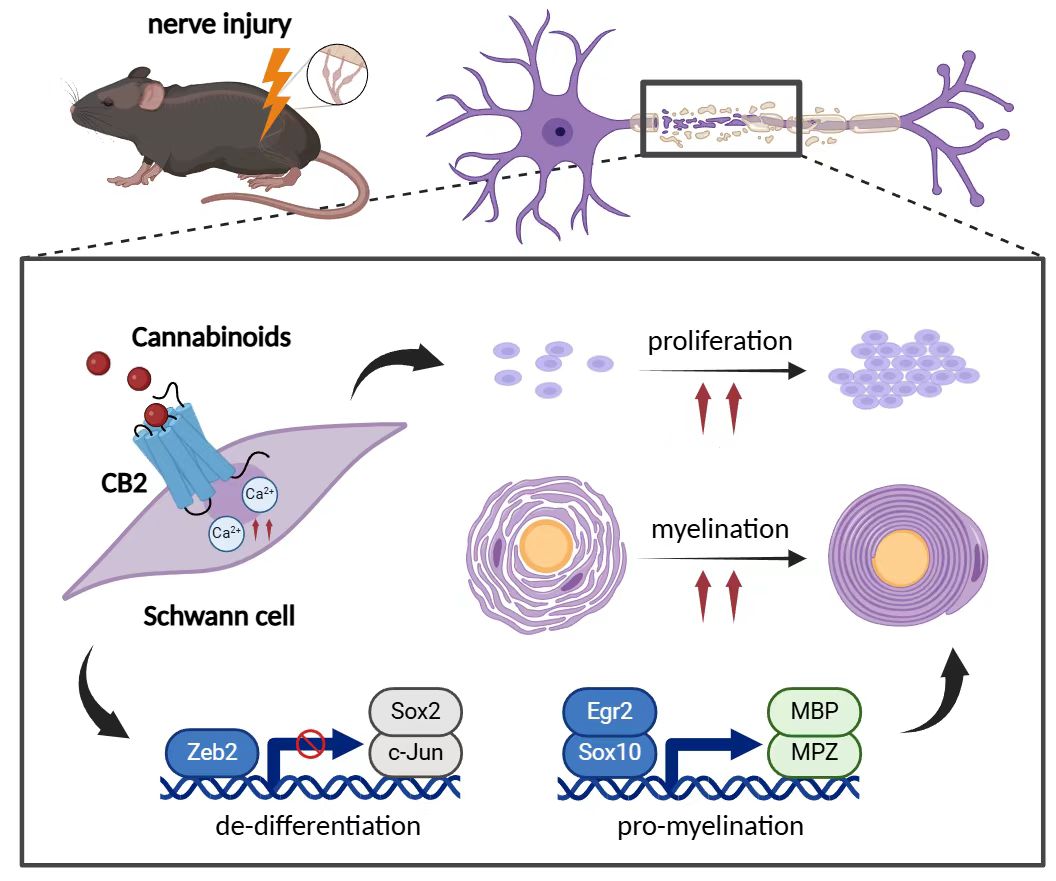


**FigS9. Schematic diagram showing the process of CB2 facilitates the SCs-mediated peripheral nerve regeneration.**
